# The Scope of Virtual Reality Simulators in Radiology Education: Systematic Literature Review

**DOI:** 10.2196/52953

**Published:** 2024-05-08

**Authors:** Shishir Shetty, Supriya Bhat, Saad Al Bayatti, Sausan Al Kawas, Wael Talaat, Mohamed El-Kishawi, Natheer Al Rawi, Sangeetha Narasimhan, Hiba Al-Daghestani, Medhini Madi, Raghavendra Shetty

**Affiliations:** 1 Department of Oral and Craniofacial Health Sciences, College of Dental Medicine, University of Sharjah, Sharjah, United Arab Emirates; 2 Department of Oral Medicine and Radiology, AB Shetty Memorial Institute of Dental Sciences, Nitte (Deemed to be University), Mangalore, India; 3 Department of Preventive and Restorative Dentistry, College of Dental Medicine, University of Sharjah, Sharjah, United Arab Emirates; 4 Department of Oral Medicine and Radiology, Manipal College of Dental Sciences, Manipal Academy of Higher Education, Manipal, India; 5 Department of Clinical Sciences, College of Dentistry, Ajman University, Ajman, United Arab Emirates

**Keywords:** virtual reality, simulators, radiology education, medical imaging, radiology, education, systematic review, literature review, imaging, meta analysis, student, students, VR, PRISMA, Preferred Reporting Items for Systematic Reviews and Meta-Analyses

## Abstract

**Background:**

In recent years, virtual reality (VR) has gained significant importance in medical education. Radiology education also has seen the induction of VR technology. However, there is no comprehensive review in this specific area. This review aims to fill this knowledge gap.

**Objective:**

This systematic literature review aims to explore the scope of VR use in radiology education.

**Methods:**

A literature search was carried out using PubMed, Scopus, ScienceDirect, and Google Scholar for articles relating to the use of VR in radiology education, published from database inception to September 1, 2023. The identified articles were then subjected to a PRISMA (Preferred Reporting Items for Systematic Reviews and Meta-Analyses)–defined study selection process.

**Results:**

The database search identified 2503 nonduplicate articles. After PRISMA screening, 17 were included in the review for analysis, of which 3 (18%) were randomized controlled trials, 7 (41%) were randomized experimental trials, and 7 (41%) were cross-sectional studies. Of the 10 randomized trials, 3 (30%) had a low risk of bias, 5 (50%) showed some concerns, and 2 (20%) had a high risk of bias. Among the 7 cross-sectional studies, 2 (29%) scored “good” in the overall quality and the remaining 5 (71%) scored “fair.” VR was found to be significantly more effective than traditional methods of teaching in improving the radiographic and radiologic skills of students. The use of VR systems was found to improve the students’ skills in overall proficiency, patient positioning, equipment knowledge, equipment handling, and radiographic techniques. Student feedback was also reported in the included studies. The students generally provided positive feedback about the utility, ease of use, and satisfaction of VR systems, as well as their perceived positive impact on skill and knowledge acquisition.

**Conclusions:**

The evidence from this review shows that the use of VR had significant benefit for students in various aspects of radiology education. However, the variable nature of the studies included in the review reduces the scope for a comprehensive recommendation of VR use in radiology education.

## Introduction

The use of technology in education helps students achieve improved acquisition of professional knowledge and practical skills [1-3]. Virtual reality (VR) is a modern technology that simulates experience by producing 3D interactive situations and presenting objects in a virtual world with spatial dimensions [4,5]. VR technology can be classified as nonimmersive or immersive [6]. In a nonimmersive VR, the simulated 3D environment is experienced through a computer monitor [6]. On the other hand, an immersive VR provides a sense of presence in a computer-generated environment, created by producing realistic sights, sounds, and other sensations that replicate a user’s physical presence in a virtual environment [6,7]. Using VR technology, a person can look about the artificial world, navigate around in it, and interact with simulated objects or items [5,8]. Due to the broad nature of VR technology, it has many applications, some of which are in the field of medicine [9,10].

The use of VR in medicine started in the 1990s when medical researchers were trying to create 3D models of patients’ internal organs [11-13]. Since then, VR use in the field of medicine and general health care has increased substantially to cover many areas including medical education. Radiology education has also come to see the use of VR technology in the recent past [14]. The use of VR in radiology education enables students to practice radiography in a virtual environment, which is radiation free [15]. Additionally, the use of VR enables effective and repeatable training. This allows trainees to recognize and correct errors as they occur [16,17]. The aim of this review is to explore the scope of VR in radiology education.

## Methods

This systematic review has been performed using the PRISMA (Preferred Reporting Items for Systematic Review and Meta-Analysis) guidelines [18] [Checklist 1]).

### Information Sources and Study Selection

The bibliographic databases used were PubMed, Scopus, ScienceDirect, and Google Scholar. A systematic literature search was conducted for articles published from database inception to September 1, 2023. Topic keywords were used to generate search strings. The search strings that were used are provided in Table 1. Only the first 10 pages of Google Scholar results were exported. The identified studies were then subjected to a study selection process. The search string for ScienceDirect was shorter because the database only allows a maximum of 8 Boolean operators, hence the sting had to be shortened. The search in PubMed was limited to the title and abstract. The searches in Scopus and ScienceDirect were limited to title, abstract, and keywords.

**Table 1.** Search strings used in the systematic review.

Database	Search string
PubMed and Scopus	(“virtual reality” OR “immersive reality” OR “simulated reality” OR simulator OR simulate) AND (radiology OR radiography OR imaging OR radiologist) AND (education OR teaching)
ScienceDirect and Google Scholar	(“virtual reality” OR “immersive reality” OR “simulated reality” OR simulator) AND (radiology OR radiography OR imaging) AND (education OR teaching)

### Inclusion and Exclusion Criteria

Original research articles written in the English language were included in the review. Studies conducted on medical, dental, and allied health sciences students (undergraduate and postgraduate) from any part of the world were included in the review. Studies exploring the use of VR learning in radiology education were included.

Narrative reviews, scoping reviews, systematic reviews, meta-analyses, editorials, and commentaries were excluded. Studies that did not align with the required study objective were excluded.

### Method of Quality Assessment

Randomized controlled trials (RCTs) and randomized experimental studies were appraised using the RoB 2 tool from the Cochrane Collaboration [19]. A visualization of the risk-of-bias assessment was done using the web-based *robvis* tool [20]. Cross-sectional studies were appraised using the appraisal checklist for analytical cross-sectional studies from the Joanna Briggs Institute [21].

### Data Extraction

Each article included in the review was summarized in a table, including basic study characteristics. The extracted attributes were study author(s), publication year, study design, type and number of participants, type of radiology education under study, and the outcome being assessed. The extracted data are provided in Table 2.

**Table 2.** Data extraction table of the studies included in the systematic review.

Study	Study design	Participants	Aspect of radiology	Study outcome
Ahlqvist et al [22]	RCT^a^	31 first-year radiologic technology student	Diagnostic radiology	Assessing radiographic image quality
Bridge et al [23]	Randomized experimental trial	48 medical imaging students	General radiology	Student satisfaction and technical skills (ie, patient positioning, equipment positioning, and mean proficiency)
Gunn et al [24]	Randomized experimental trial	45 medical imaging student	Diagnostic radiology	Technical radiographic skills
Gunn et al [25]	Cross-sectional study	28 medical imaging students and 38 radiation therapy students	Interventional radiology	Students’ perceived confidence in performing diagnostic and planning CT^b^ scans
Jensen et al [26]	Cross-sectional study	10 radiography students	General radiology	Self-perceived clinical readiness of radiography students regarding the acquisition of wrist radiographs
Kato et al [27]	Randomized experimental trial	30 first-year radiologic technology student	General radiology	Radiographic skills proficiency
Nilsson et al [28]	Randomized experimental trial	57 dental students	Oral radiology	Interpretation of spatial relations in radiographs using parallax
Nilsson et al [29]	Randomized experimental trial	45 dental students	Oral radiology	Interpretation of spatial relations in radiographs using parallax
O’Connor and Rainford [30]	Randomized experimental trial	191 radiography students	General radiology	Patient preparation, room preparation, patient care, radiographic technique, and image appraisal
O’Connor et al [15]	Cross-sectional study	105 first-year radiography students	General radiology	Reporting student experience
Rainford et al [31]	Cross-sectional study	35 radiography students and 100 medical students	Interventional radiology	Reporting student experience
Rowe et al [32]	Randomized experimental trial	188 radiography students	General radiology	Technical skills (ie, duration of the exam, frequency of machinery movement, frequency of incorrect machinery movement, frequency of radiographic exposure errors, and frequency of patient positioning errors)
Sapkaroski et al [33]	Cross-sectional study	92 medical radiation science students	General radiology	Reporting student experience
Sapkaroski et al [34]	RCT	76 first-year radiography students	Radiation technology	Patient positioning
Sapkaroski et al [35]	RCT	76 radiography students	General radiology	Students’ perception about developing radiographic hand positioning skills.
Shanahan [36]	Cross-sectional study	86 first-year radiography students	General radiology	Reporting student perception
Wu et al [37]	Cross-sectional study	18 medical students	General radiology	Reporting student perception

a RCT: randomized controlled trial.

b CT: computed tomography.

## Results

### Search Results

**Figure 1.**
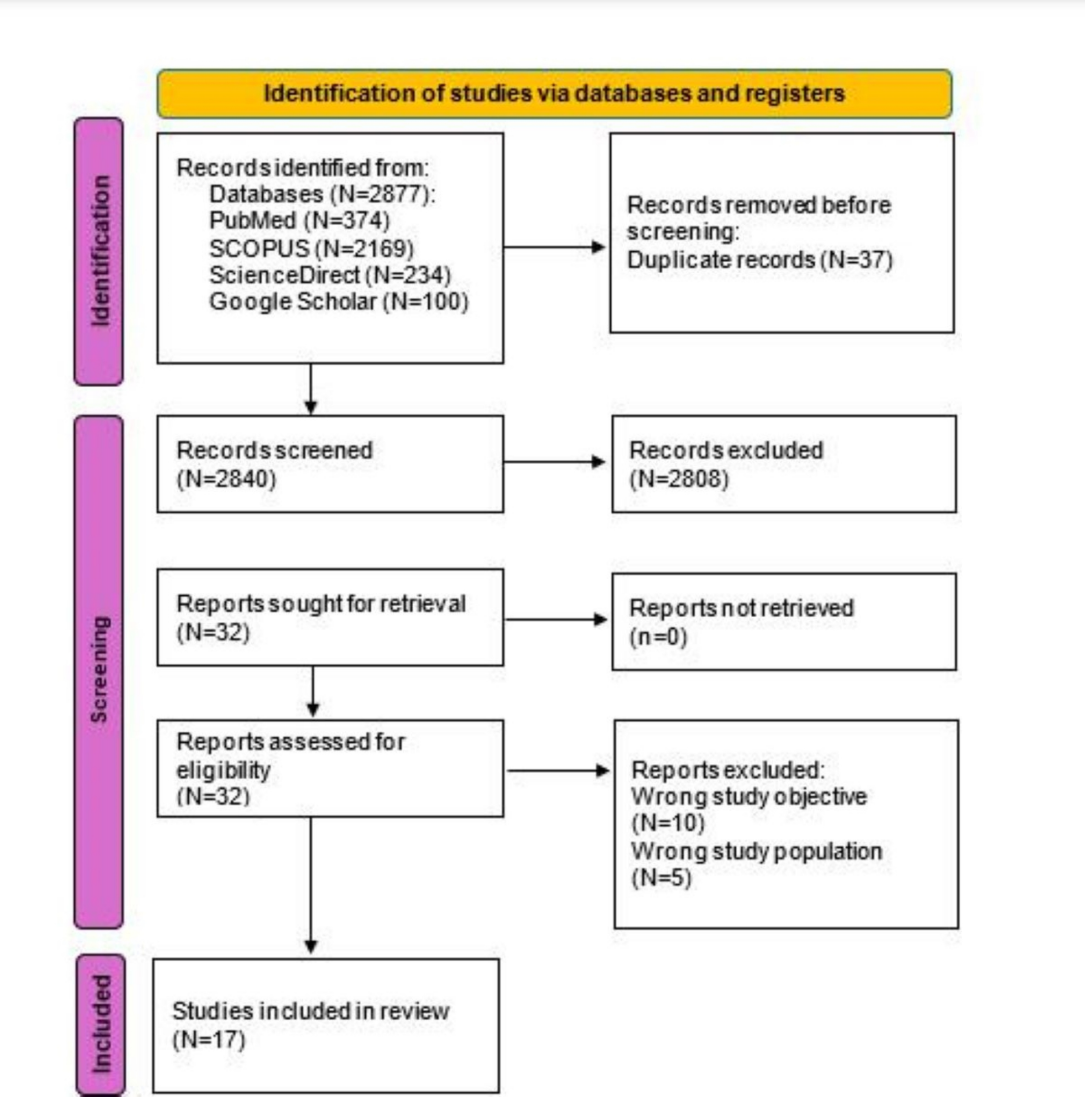
PRISMA (Preferred Reporting Items for Systematic Reviews and Meta-Analyses) flowchart showing the study selection process.

The database search identified a total of 2877 studies; 374 (13%) studies were from PubMed, 2169 (75.4%) were from Scopus, 234 (8.1%) were from ScienceDirect, and 100 (3.5%) were from Google Scholar. Before the screening procedure, 37 duplicates were removed. During title and abstract screening, 2808 articles were excluded since they did not align with the eligibility criteria. The remaining 32 articles were then subjected to a full-text review, and 15 were excluded for reasons provided in Figure 1, which shows the study selection process [38]. At the end of the process, 17 studies were found eligible for inclusion in the review.

### Characteristics of Included Studies

Among the 17 studies, 3 (18%) RCTs, 7 (41%) randomized experimental trials, and 7 (41%) cross-sectional studies were included. The studies encompassed various aspects of radiology education, including dental radiology [28,29], diagnostic radiology [22,24], and interventional radiology [25,31].

### Results of Quality Assessment

Among the 7 cross-sectional studies, 2 (29%) scored “good” in overall quality and the remaining 5 (71%) scored “fair.” The results for the quality appraisal of cross-sectional studies are shown in Table 3. Studies were appraised using the checklist for analytical cross-sectional studies from the Joanna Briggs Institute [21].

**Figure 2.**
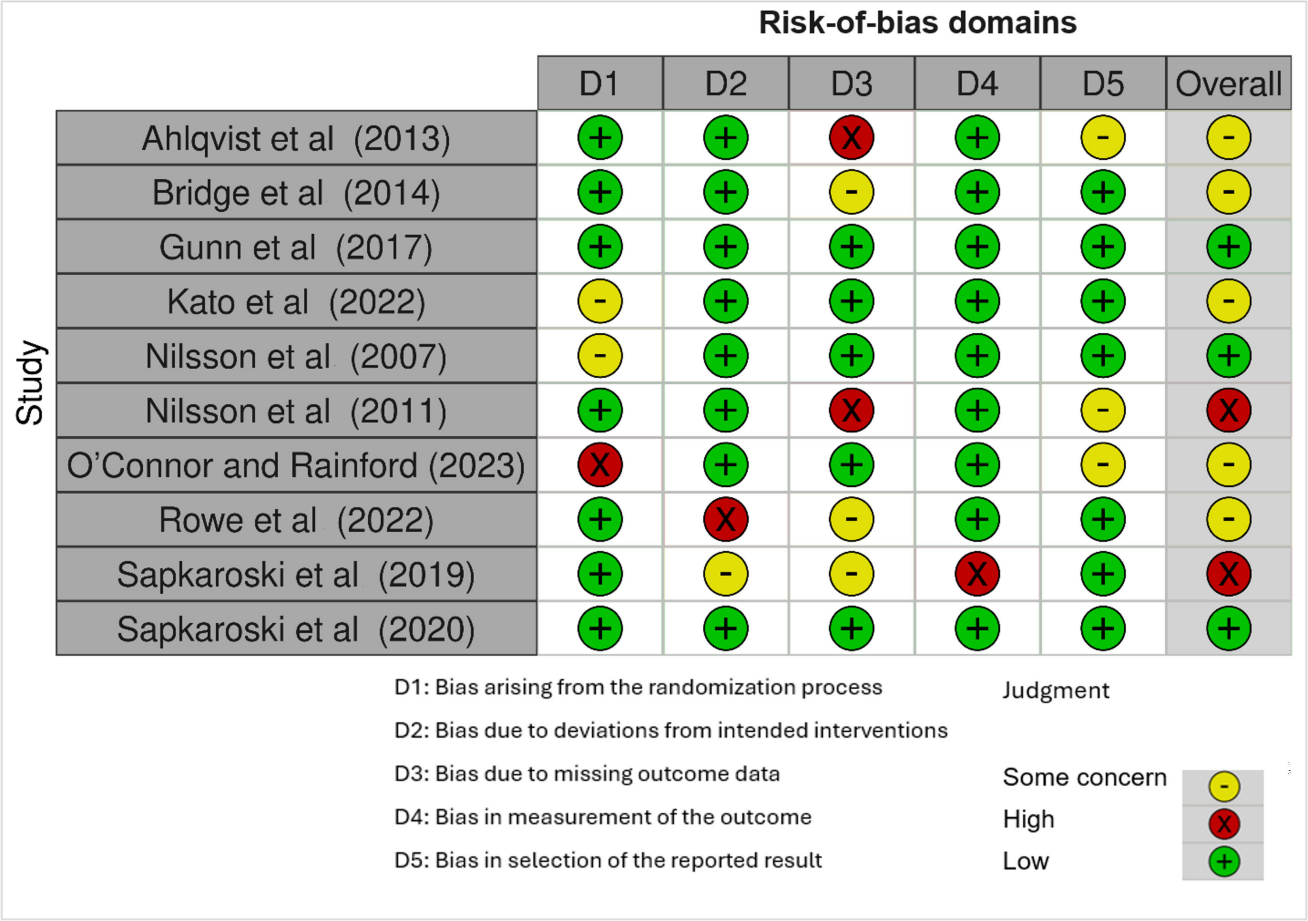
Risk-of-bias graph using a traffic light plot for different domains (D1 to D5) [22-24,27-30,32,34,35].

**Figure 3.**
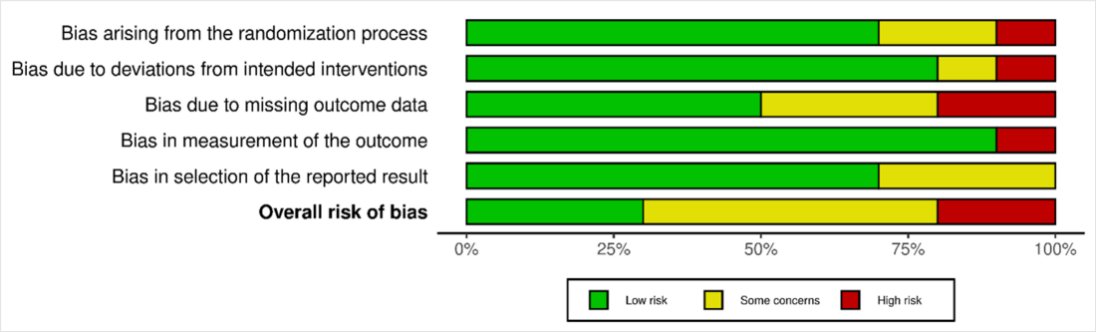
Weighted bar plots displaying the distribution of risk-of-bias judgments within each bias domain.

Among the 10 randomized trials, 3 (30%) had a low risk of bias, 5 (50%) showed some concerns, and 2 (20%) had a high risk of bias. These results are shown in Table 4. RCTs were appraised using the RoB 2 tool from the Cochrane Collaboration [19]. A risk-of-bias graph (Figure 2) and a risk-of-bias summary (Figure 3) are also provided.

**Table 3.** Appraisal for cross-sectional studies included in the systematic review.

Study	Item 1^a^	Item 2^b^	Item 3^c^	Item 4^d^	Item 5^e^	Item 6^f^	Item 7^g^	Item 8^h^	Overall quality
Gunn et al [25]	Yes	Yes	Yes	Yes	Yes	No	Yes	Yes	Good
Jensen et al [26]	Yes	Yes	Yes	No	No	N/A^i^	Yes	Yes	Fair
O’Connor et al [15]	Yes	Yes	Yes	Yes	No	N/A	Yes	No	Fair
Rainford et al [31]	Yes	Yes	Yes	Yes	No	N/A	Yes	Yes	Fair
Sapkaroski et al [33]	No	Yes	Yes	Unclear	No	N/A	Yes	Unclear	Fair
Shanahan [36]	No	Yes	Unclear	Yes	Yes	Unclear	Yes	Yes	Good
Wu et al [37]	Yes	Yes	Yes	Yes	No	N/A	Yes	Yes	Fair

a Item 1: were the criteria for inclusion in the sample clearly defined?

b Item 2: were the study subjects and the setting described in detail?

c Item 3: was the exposure measured in a valid and reliable way?

d Item 4: were objective, standard criteria used for measurement of the condition?

e Item 5: were confounding factors identified?

f Item 6: were strategies to deal with confounding factors stated?

g Item 7: were the outcomes measured in a valid and reliable way?

h Item 8: was appropriate statistical analysis used?

i N/A: not assessable.

**Table 4.** Risk-of-bias assessment for randomized trials included in the systematic review.

Study	D1^a^	D2^b^	D3^c^	D4^d^	D5^e^	Overall
Ahlqvist et al [22]	Low	Low	High	Low	Some concerns	Some concerns
Bridge et al [23]	Low	Low	Some concerns	Low	Low	Some concerns
Gunn et al [24]	Low	Low	Low	Low	Low	Low
Kato et al [27]	Some concerns	Low	Low	Low	Low	Some concerns
Nilsson et al [28]	Some concerns	Low	Low	Low	Low	Low
Nilsson et al [29]	Low	Low	High	Low	Some concerns	High
O’Connor and Rainford [30]	High	Low	Low	Low	Some concerns	Some concerns
Rowe et al [32]	Low	High	Some concerns	Low	Low	Some concerns
Sapkaroski et al [34]	Low	Some concerns	Some concerns	High	Low	High
Sapkaroski et al [35]	Low	Low	Low	Low	Low	Low

a D1: risk of bias arising from the randomization process.

b D2: risk of bias due to deviations from the intended interventions (effect of assignment to intervention).

c D3: risk of bias due to missing outcome data.

d D4: risk of bias in measurement of the outcome.

e D5: risk of bias in selection of the reported result.

### Type of VR Hardware and Software Used in the Studies

The studies used a wide range of VR software and hardware. Some of the studies used 3D simulation software packages displayed on 2D desktop computers [22,24,25,36], whereas others used headsets for an immersive VR environment [15,23,26,35,37]. The most used VR teaching software were the CETSOL VR Clinic software [33,35], Virtual Medical Coaching VR software [15,30,32], Projection VR (Shaderware) software [36], SieVRt VR system (Luxsonic Technologies) [37], medical imaging training immersive environment software [23], VR CT Sim software [25], VitaSim ApS software [26], VR X-Ray (Skilitics and Virtual Medical Coaching) software [27], and radiation dosimetry VR software (Virtual Medical Coaching Ltd) [31].

### Effect of VR Teaching on Skill Acquisition

Ahlqvist et al [22] looked at how virtual simulation can be used as an effective tool to teach quality assessment of radiographic images. They also compared how it faired in comparison to traditional teaching. The study reported a statistically significant improvement in proficiency from before training to after training. Additionally, the study reported that the proficiency score improvement for the VR-trained students was higher than that for the students trained using conventional method.

In the study conducted by Sapkaroski et al [34], students in the VR group demonstrated significantly better patient positioning skills compared to those in the conventional role-play group. The positioning parameters that were assessed were digit separation and palm flatness (the VR group scored 11% better), central ray positioning onto the third metacarpophalangeal joint (the VR group scored 23% better), and a control position projection of an oblique hand. The results for the control position projection indicated no significant difference in positioning between the 2 groups [34].

Bridge et al [23] also performed a performance comparison between students trained by VR and traditional methods. They assessed skills about patient positioning, equipment positioning, and time taken to complete a performative role-play. Students in the VR group performed better than those in the control group, with 91% of them receiving an overall score of above average (>3). The difference in mean group performance was statistically significant (*P*=.0366). Similarly, Gunn et al [24] reported improved and higher role-play skill scores for students trained using VR software simulation compared to those trained on traditional laboratory simulation. The mean role-play score for the VR group was 30.67 and that for the control group was 28.8 [24].

Another study reported that students trained using VR performed significantly better (ranked as “very good” or “excellent”) than the control group (conventional learning) in skills such as patient positioning, selecting exposure factors, centering and collimating the x-ray beam, placing the anatomical marker, appraisal of image quality, equipment positioning, and procedure explanation to the patient [30]. Another recently conducted study found that the VR-taught group achieved better test duration and fewer errors in moving equipment and positioning a patient. There was no significant difference in the frequency of errors in the radiographic exposure setting such as source-to-image distance between the VR and the physical simulation groups [32].

Nilsson et al [28] developed a test to evaluate the student’s ability to interpret 3D information in radiographs using parallax. This test was applied to students before and after training. There was a significantly larger (*P*<.01) pre-post intervention mean score for the VR group (3.11 to 4.18) compared to the control group (3.24 to 3.72). A subgroup analysis was also performed, and students with low visuospatial ability in the VR group had a significantly higher improvement in the proficiency test compared to those in the control group. The same authors conducted another follow-up study to test skill retention [29]. Net skill improvement was calculated as the difference in test scores after 8 months. The results from the proficiency test showed that the ability to interpret spatial relations in radiographs 8 months after the completion of VR training was significantly better than before VR training. The students who trained conventionally showed almost the same positive trend in improvement. The group difference was smaller and not statistically significant. This meant that, 8 months after training, the VR group and the traditionally trained group had the same skill level [29].

Among the included studies, only 1 reported that the VR group had lower performance in proficiency tests and radiographic skill tests, compared to a conventionally trained group. The study, conducted in 2022, showed that the proficiency of the VR group was significantly lower than that of the conventional technique group in performing lateral elbow and posterior-anterior chest radiography [27]. An itemized rubric evaluation used in the study revealed that the VR group also had lower performance in most of the radiographic skills, such as locating and centering of the x-ray beam, side marker placement, positioning the x-ray image detector, patient interaction, and process control and safety [27]. The study concluded that VR simulation can be less effective than real-world training in radiographic techniques, which requires palpation and patient interaction. These results may be different from those of other studies due to different outcome evaluation methods and since they used head-mounted display VR coaching, whereas the other studies, except O’Connor et al [15], used VR on a PC monitor.

All of the studies except Kato et al [27] agreed that VR use was more effective for students in developing radiographic and radiologic skills. Despite this general agreement, there were slight in-study variations in learning outcomes, which made some of the studies look at factors that may influence skill and knowledge acquisition during VR use. In studies such as Bridge et al [23], it was noted that the arrangement of equipment had the greatest influence on the overall score. After performing a multivariable analysis, Gunn et al [24] reported that there was no effect of age, gender, and gaming skills or activity on the outcome of VR learning. In the study by Shanahan [36], a few students (19/84, 23%) had previously used VR simulation software. This had no bearing on the learning outcomes. Another observation in the same study was that student age was found to significantly affected the student’s confidence about skill acquisition after VR training [36].

### Students’ Perception of VR Uses for Learning

The findings from the study by Gunn et al [25] revealed that 68% of students agreed or strongly agreed that VR simulation was significantly helpful in learning about computed tomography (CT) scanning. In another study by Jensen et al [26], 90% of the students strongly agreed that VR simulators could contribute to learning radiography, with 90% reporting that the x-ray equipment in the VR simulation was realistic. In the study by Wu et al [37], most of the students (55.6%) agreed or somewhat agreed that VR use was useful in radiology education. Similarly, 83% of the students in Shanahan’s [36] study regarded VR learning with an ease of use. In the same study, students also reported that one of the major benefits of VR learning include using the simulation to repeat activities until being satisfied with the results (95% of respondents). Students also stated that VR enabled them to quickly see images and understand if changes needed to be made (94%) [36]. In the study by Gunn et al [25], 75% of medical imaging students agreed on the ease of use and software enjoyment in VR simulated learning. In the same study, 57% of the students reported a positive perceived usefulness of VR. Most respondents (80%) in the study by Rainford et al [31] favored the in-person VR experience over web-based VR. Similarly, 58% of the respondents in the study conducted by O’Connor et al [15] reported enjoying learning using VR simulation. In the study by Wu et al [37], 83.3% of students agreed or strongly agreed that they enjoyed using VR for learning. Similarly, the studies by Rainford et al [31] and O’Connor et al [15] reported student recommendation of 87% and 94%, respectively, for VR as a learning tool.

### Students’ Perceived Skill and Knowledge Acquisition

In the study by Bridge et al [23], students who trained using VR reported an increase in perceived skill acquisition and high levels of satisfaction. The study authors attributed this feedback to the availability of “gold standards” that showed correct positioning techniques, as well as instant feedback provided by the VR simulators. Gunn et al [25] examined students’ confidence in performing a CT scan in a real clinical environment after using VR simulations as a learning tool. The study reported an increase (from before to after training) in the students’ perceived confidence in performing diagnostic CT scans. Similarly, the study by Jensen et al [26] reported that the use of VR had influenced students’ self-perceived readiness to perform wrist x-ray radiographs. The study, however, found no significant difference in pre- and posttraining (perceived preparedness) scores. The pre- and posttraining scores were 75 (95% CI 54-96) and 77 (95% CI 59-95), respectively. The study by O’Connor et al [15] looked at the effect of VR on perceived skill adoption. Most of the students in the study reported high levels of perceived knowledge acquisition in the areas of beam collimation, anatomical marker placement, centering of the x-ray tube, image evaluation, anatomical knowledge, patient positioning, and exposure parameter selection to their VR practice. However, most students felt that VR did not contribute to their knowledge of patient dose tracking and radiation safety [15]. In the study by Rainford et al [31], 73% of radiography and medical students felt that VR learning increased their confidence across all relevant learning outcomes. The biggest increase in confidence level was regarding their understanding of radiation safety matters [31]. Sapkaroski et al [33] performed a self-perception test to see how students viewed their clinical and technical skills after using VR for learning. In their study, students reported a perceived improvement in their hand and patient positioning skills. Their study also compared 2 software, CETSOL VR Clinic and Shaderware. The cohort who used CETSOL VR Clinic had higher scores on perceived improvement [33]. Sapkaroski et al [35] compared the student’s perception scores on the educational enhancement of their radiographic hand positioning skills, after VR or clinical role-play scenario training. Although the VR group scored higher, there was no significant difference between the scores for the 2 groups [35]. In the study by Shanahan [36], when the perception of skill development was evaluated, most of the students reported that the simulation positively developed their technical (78%), radiographic image evaluation (85%), problem-solving (85%), and self-evaluation (88%) abilities. However, in the study by Kato et al [27], there was no difference in the perceived acquisition of knowledge among students using traditional teaching and VR-based teaching.

## Discussion

### Principal Findings

The results presented in this review reveal strong evidence for the effectiveness of VR teaching in radiology education, particularly in the context of skill acquisition and development [22,24,27,30,32,34].

In this review, quality appraisal of the cross-sectional studies revealed that the strategies for deal with confounding factors was one of the factors directly affecting the reliability of the results. Similarly, the appraisal of the randomized trials revealed that the bias arising due to missing outcome data was one of the factors directly affecting the reliability of the results.

All the studies found that VR-based teaching had a positive impact on various areas of radiographic and radiologic skill development. In comparison to the traditional way of teaching, only 1 study by Kato et al [27] reported VR teaching as inferior to traditional teaching. The studies consistently reported better improvements in proficiency, patient positioning outcomes, equipment handling, and radiographic techniques among students trained using VR. According to Nilsson et al [29], O’Connor et al [15], and Wu et al [37], the improvements were due to the immersive and interactive nature of VR simulations, which allowed learners to engage with radiological scenarios in a dynamic and hands-on manner. The studies also revealed that VR learning has the ability to easily and effectively introduce students to new skills. It was also found that existing skills could be improved, mainly through simulation feedback that happens in real time during training [22,24,28,30,36].

The improvement of skills after VR training have been noted in different domains, including patient positioning, equipment positioning, equipment knowledge, assessment of radiographic image quality, and patient interaction. Improvement was also observed in other skills such as as central ray positioning, source-to-image distance, image receptor placement, and side marker placement [22,24,30,32,34]. Two studies, Nilsson et al [28] and Nilsson et al [29], looked at how VR affected the students’ ability to interpret 3D information in radiographs using parallax. They both reported a positive effect. Nilsson et al [29] also gave insights into the long-term benefits of VR training in radiology. Eight months after training, the control (traditionally taught) group in Nilsson et al [29] showed a slight increase in skills, but the VR-trained group still maintained a significantly higher skill level. This finding shows the enduring impact of VR-based education on skill acquisition in radiology. Although most studies supported the effectiveness of VR in radiology education, 1 study reported contrasting results [27]. VR-trained students were found to perform worse than traditionally trained students in conducting lateral elbow and posterior-anterior chest radiography in Kato et al [27]. This difference in results was, according to the authors, attributed to the use of a different rubric evaluation method and the use of a head-mounted display–based immersive VR system, which was not used in other studies. These 2 reasons may be the reason for the variation in study findings.

A wide range of VR software with different functions were used in the studies. In addition to acquiring radiographic images, the CETSOL VR Clinic software facilitated students to interact with their learning environment [33,35]. Students using the Virtual Medical Coaching VR software performed imaging exercise on a virtual patient with VR headsets and hand controllers [15,30,32]. The SieVRt VR system displayed Digital Imaging and Communications in Medicine format images in a virtual environment, thus facilitating teaching [37]. The medical imaging training immersive environment simulation software provided automated feedback to the learners including a rerun of procedures, thus highlighting procedural errors [23]. The VR CT Sim software allowed the student virtually to perform the complete CT workflow [25]. Students could manipulate patient positioning and get feedback from the VitaSim ApS software [26]. The VR X-Ray software allowed students to manipulate radiographic equipment and patient’s position with a high level of immersive experience [27]. Radiation dosimetry VR software facilitated virtual movement of the staff and equipment to radiation-free areas, thus optimizing radiation protection [31].

The included studies also looked at factors that could influence skill acquisition when VR is used in radiology education. Bridge et al [23], Gunn et al [24], Kato et al [27], and Shanahan [36] investigated factors such as age, gender, prior gaming experience, and familiarity with VR technology. However, these factors were shown to have no significant effect on VR learning outcomes. This shows that VR education can equally accommodate a wide range of learners, regardless of experience or existing attributes.

Across several studies, positive feedback emerged regarding the utility, ease of use, enjoyment, and perceived impact on skill and knowledge acquisition. The included studies consistently reported positive perceptions of VR use among students [25,26,37]. Gunn et al [25] reported that a significant proportion of medical imaging and radiation therapy students found the use of VR simulation to be significantly helpful in learning about CT scanning. Similarly, Jensen et al [26] and Wu et al [37] reported that a majority of students agreed on the usefulness of VR in radiology education. Another aspect that received positive feedback was the ease of use. Students liked the ability to repeat tasks until they were satisfied with the results and the ability to quickly visualize radiographs to determine the need for revisions [36]. Rainford et al [31] and O’Connor and Rainford [30] found that most students would recommend VR as a learning tool to other students.

Several studies investigated student’s perceptions of skill and knowledge acquisition when using VR for radiology education. Bridge et al [15] and O’Connor et al [23] discovered an increase in students’ perceived acquisition of radiographic skills. Gunn et al [25] reported an increase in students’ perceived confidence to perform CT scans after learning using VR simulations. According to Rainford et al [31], a large percentage of radiography and medical students felt that VR learning boosted their confidence across all relevant learning outcomes, with the highest levels of confidence recorded in radiation safety. Sapkaroski et al [33] discovered that after using VR for learning, students experienced an improvement in their hand and patient placement skills. In summary, the positive feedback from the students shows that VR use in radiology education is a useful, engaging, and effective teaching tool. This perceived acquisition of skills is backed by the results from the proficiency tests.

The VR modalities used in some of the studies allowed remote assistance from an external agent (teacher), as the VR training is conducted in front of a screen while being part of a team, with the teacher making constant corrections and indications [22,24,27]. However, researchers are looking into VR systems with artificial intelligence–supported tutoring, which includes the assessment of learners, generation of learning content, and automated feedback [39].

### Conclusion

Findings from the included studies show that VR-based teaching offers substantial benefits in various aspects of radiographic and radiologic skill development. The studies consistently reported that students educated using VR systems improved significantly in overall proficiency, patient positioning, equipment knowledge, equipment handling, and radiographic techniques. However, the variable nature of the studies included in the review reduces the scope for a comprehensive recommendation of VR use in radiology education. A key contributing factor to relatively better learning outcomes was the immersive and interactive nature of VR systems, which provided real-time feedback and dynamic learning experiences to students. Factors such as age, gender, gaming experience, and familiarity with VR systems did not significantly influence learning outcomes. This shows that VR can be used for diverse groups of students when teaching radiology. Students generally provided positive feedback about the utility, ease of use, and satisfaction of VR, as well as its perceived impact on skill and knowledge acquisition. These students’ reports show the value of VR as an important, interesting, and effective tool in radiology education.

## Supplementary material

10.2196/52953 Checklist 1 PRISMA (Preferred Reporting Items for Systematic Reviews and Meta-Analyses) checklist.

## Abbreviations

CT: computed tomography
PRISMA: Preferred Reporting Items for Systematic Reviews and Meta-Analyses
RCT: randomized controlled trial
VR: virtual reality
